# Treatment Success and User-Friendliness of An Electric Toothbrush App: A Pilot Study

**DOI:** 10.3390/dj8030097

**Published:** 2020-09-01

**Authors:** Viviane Humm, Daniel Wiedemeier, Thomas Attin, Patrick Schmidlin, Stefanie Gartenmann

**Affiliations:** 1 Clinic of Conservative and Preventive Dentistry, Center of Dental Medicine, University of Zurich, 8032 Zurich, Switzerland; viviane.humm@hotmail.ch (V.H.); thomas.attin@zzm.uzh.ch (T.A.); Patrick.Schmidlin@zzm.uzh.ch (P.S.); 2 Statistical Services, Center of Dental Medicine, University of Zurich, 8032 Zurich, Switzerland; daniel.wiedemeier@zzm.uzh.ch

**Keywords:** compliance, mobile application, smartphones, index, dental plaque

## Abstract

Electronic and mobile health (eHealth/mHealth) are rapidly growing areas in medicine and digital technologies are gaining importance. In dentistry, digitalization is also an emerging topic, whereby more and more applications are being offered. As an example, using real-time feedback, digital application software (an app) was designed to help users brush their teeth more accurately. However, there is no data on the effectiveness and haptic of such apps. Therefore, a single-blinded, randomized controlled clinical trial was designed: twenty volunteers received an electric toothbrush with an associated app to assess whether the app-assisted toothbrushing is better than without. After a short period of familiarization with the electric toothbrush, plaque index (O‘Leary et al. 1972) was recorded and subjects were assigned to the test (with app; n = 10) or the control group (no app; n = 10). At the end of the 2-week pilot study period, plaque was again assessed and participants in the test group completed a questionnaire about the app’s user-friendliness. Statistical analysis revealed no significant differences between the test and control groups. The plaque index improved on average by 8.5% points in the test and 4.7% points in the control group. Fifty percent of the test group participants were of the opinion that they had achieved better cleaning results and would recommend the app to others, although the app contributed only marginally to increased plaque removal. However, such apps may nevertheless be helpful as motivational tools, especially when tracking and monitoring cleaning data. Therefore, more development and research on this topic is indicated.

## 1. Introduction

Experimental gingivitis studies from the 1960s clearly show a link between plaque and inflammation and that if left untreated, biofilm accumulation leads to further tissue destruction [1]. This underlines the importance of effective and reliable home care regimens, using toothbrushes and other site-specific aids, that assist patients in cleaning their teeth thoroughly, frequently and reliably [2]. Particularly in regard to gingivitis, power-driven toothbrushes have proven to be more beneficial than manual toothbrushes [3]. Given that two minutes of electric tooth brushing is equivalent to six minutes of manual tooth brushing, it is not surprising that electric toothbrushes have gained popularity in maintaining oral health [4,5].

At the same time, mobile computing devices such as smartphones and tablets have been launched with new technologies, which incorporate communication and computing features [6]. Apps have been developed and have evolved which influence our daily life, communication and education, even expanding into medical practice and science [7]. An increasing number of healthcare professionals, medical students and patients already utilize a plethora of healthcare apps for smartphones [8].

One of the many indications for smartphone app usage in healthcare is patient education. Whether it is for the training of healthcare professionals, communication between patient and clinician or patient self-use, apps are thought to simplify and improve patient care [8,9]. An informed patient may assume greater personal responsibility, leading to better compliance and therefore a higher success rate in disease management [10]. Technology-assisted education may improve patient compliance among a variety of different ages and educational levels as well. In particular, visualization and digital animation are powerful tools in motivating patients to take responsibility and action in health care issues [9,11]. Motivational apps, for example, may inspire patients to approach oral home-care measures in ways unreached to date. In dentistry, periodontitis, gingivitis and caries are the most frequent bacteria-based diseases [12], in the case of caries, a correlation between salivary inflammatory cytokines, such as interleukin (IL-6) were found [13]. Both are preventable and, in the case of gingivitis, reversible, through proper home care.

As a consequence, many dental problems can be avoided simply by optimal plaque control. Therefore, it is particularly important that patients actively participate in oral hygiene maintenance. Often, without frequent recall appointments, home-care instructions are forgotten and over time many patients become negligent. With the help of an app, a patient and the dentist or dental hygienist may gain insight into the patient’s behavior between office visits. Further, electronic reminders and easy-to-call-up explanations may help the patient to implement medical recommendations, and in this case, oral hygiene measures needed to attain and maintain oral health.

These assumptions, however, remain theoretical. An important question to be answered remains, namely whether apps provide actual health benefits to patients. Therefore, this pilot study was undertaken to determine whether a smartphone app for use with an electrical oscillating toothbrush improves plaque removal, increases user compliance and may be considered user-friendly.

## 2. Materials and Methods

### 2.1. Patient Population

Twenty healthy lay-people in Zurich, with no professional association to dentistry, were asked to participate in this study. The subjects, over 18 years of age with a smartphone, were randomly recruited from the investigator’s circle of acquaintances. All subjects had at least 24 teeth present and had neither undergone dental or dental hygiene treatment in the last two weeks before the study began, nor during the duration of the study. Exclusion criteria were applied to subjects with fixed braces or partial dentures with less than 24 existing teeth, minors and potential participants without a smartphone. All participants had to be able to brush their teeth independently. Seventeen females and 3 males with ages ranging from 21 to 32 were included. Prior to the pilot study, participants cleaned their teeth with either a manual toothbrush, a sonic toothbrush or a rotating toothbrush. For this study, all participants were given an oscillating–rotating toothbrush (Oral B Genius 9000 Procter and Gamble, Cincinnati, OH, USA), which was then used exclusively for the 2 + 2-week study duration.

This study was reviewed and approved by the Canton Zurich Ethics Review Board (BASEC-ID: 2017-01718, 15 January 2018) and registered on the German Clinical Trials Register (DRKS00013560, registration date 01 March 2018). All study participants were informed of the purpose and scope of the study and provided written consent. Participants were also assured that the data collected would be anonymized.

### 2.2. Experimental Procedure

Patients were given a two-week familiarization period with an electrical toothbrush. No formal training as to best use practices of the toothbrush was provided. The study was designed so that the test subjects would rely on the app for guidance in optimal tooth cleaning. They were, however, advised to brush their teeth twice a day. Plaque indices (O‘Leary et al. 1972) were recorded by a single operator applying a plaque indicator (Paro Plak, Esro AG, Kilchberg, Switzerland), which marked the beginning of the study (baseline). Subjects were then randomly divided into two groups (Random Sequence Generator, random.org). The experimental group consisting of ten volunteers was asked to use an app developed for this particular electric toothbrush and the control group (n = 10) was instructed to use only the electric toothbrush without the app. No professional instruction for using the app was provided, as it is propagated as self-explanatory. At the end of the two-week observation period, the plaque index was measured again.

### 2.3. The App

The smartphone app (Oral B Genius 9000 Procter and Gamble, Cincinnati, OH, USA), in short, records the tooth brushing behavior, transmits it to the app via Bluetooth technology, which is then analyzed by a software. The app provides real-time feedback on the cleaning duration of the individual tooth sextants. Position recognition allows the user to see which tooth surfaces have been brushed and for how long. The app can be modified and adjusted to the patient’s individual needs.

### 2.4. Questionnaire

After recording the final plaque index, the teeth were professionally cleaned and the subjects in the test group completed a questionnaire about the user-friendliness of the app. Questions asked concerned the toothbrush used prior to the study and if the app was useful. Participants were able to comment on subjectively perceived cleaning quality with the app and if they will continue its use and/or recommend it to others (Appendix A).

### 2.5. Statistical Analysis

The primary endpoint chosen was the difference in plaque index change (∆) between the intervention group that cleaned with the app and the control group that cleaned without the app, as assessed by a Wilcoxon rank-sum test. Moreover, the ∆ plaque index change between the start and the end of the study was assessed for both groups individually, using a Wilcoxon signed-rank test. All analyses were performed with the software package R [14]. The significance level alpha was chosen to be 0.05. Descriptive statistics (mean and SD) were calculated for all groups.

## 3. Results

**Figure 1:**
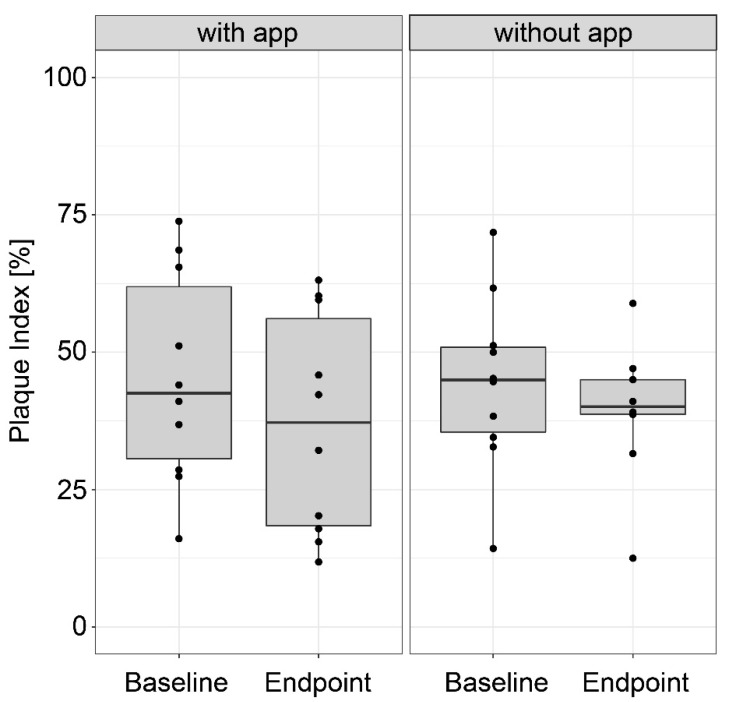
Box plot representations of the plaque index in the group that cleaned with the app and the group that cleaned without the app (n = 10 per group), at the start and the end of the study, respectively.

No relevant difference was found between the test and control groups with respect to ∆ PI (the difference in the change between the two treatments), which amounted to 3.8% points (Figure 1, *p* = 0.39). Overall, both groups had improved plaque removal values, though only slightly. The intervention group improved by 8.5% (*p* = 0.10) as compared to the control group by 4.7% (*p* = 0.56). Notably, the variability was rather greater in the intervention group than in the control group.

In the questionnaire, 9 of the 10 participants who used the app indicated that the app was understandable to them and four participants noted a better cleaning result with the app. Six participants could imagine using the app in the future and five participants would even recommend the app to others. It was positively noted that the time that was displayed when brushing the teeth and thus motivated to brush longer. Most of the participants stated that they had brushed their teeth longer with the app. Further, the molars and the inner surfaces of the teeth were brushed more consciously, and all surfaces were cleaned for the same length of time. However, the app was not consistently used by all participants, with technical difficulties being mentioned, e.g., that the sensor did not always function perfectly. In addition, there was criticism that positioning the smartphone took a lot of time. For the majority of the participants, the app was therefore not sufficiently suitable for everyday use, as time was often lacking, especially in the morning.

## 4. Discussion

In accordance with the selection criteria described, 20 subjects were included in the study from March to May 2018. Overall, there was an improvement in plaque removal in the test group as well as in the control group. Both groups had similar baseline values and no statistically significant change in plaque index between the groups could be observed.

Although there was no statistically significant improvement in plaque scores between the groups, the influence of the app on the improvement of the plaque index of almost 10% in the test group is considered to be clinically relevant if not a statistically significant result. Nevertheless, the improvement is noticeable and indicates a direction for future investigations.

The questionnaire showed that only half of the participants felt that the app helped them achieve a better result and would recommend the app to others. This result is striking because most of the participants actually achieved a somewhat better result with the app. However, only a narrow majority can imagine using the app in the future, although not every day. Since patients were questioned about the use of a toothbrush product, a self-reported bias should be recognized as a limitation.

One of the major shortcomings of this pilot study is the small number of participants. A pilot study is the first step of a research protocol and usually presents a smaller-sized study, which helps in the planning and modification of the main study [14,15]. Further, the materials needed to conduct this study (electric toothbrushes) were purchased by the university. This protocol of a strict distancing from the manufacturer ensured that a potential source of bias and/or conflict of interest could be excluded. Therefore, a pilot study was needed to obtain the information necessary for determining the number of subjects needed for a future full study.

An earlier study carried out with an oscillating–rotating toothbrush and a remote display technology (“SmartGuide”) demonstrated an extended brushing time of 38.9% compared to a manual toothbrush [16]. The same group evaluated the benefit of a pressure control using the same technology and showed that a visual display of the time and location of the toothbrush, under excessive pressure and an acoustic signal, led the participants to significantly decrease the pressure put on the toothbrush [17]. Digital guides can, therefore, be considered an important adjunct in maintaining gingival health and preventing gingival recession caused by mechanical force.

The more targeted suggestions and feedback the app provides, the more opportunities there are for improvement. Often, people are not aware of the mistakes they make when brushing their teeth. These studies show the possibility of achieving an improvement in cleaning behavior by pointing out mistakes and by providing guidance when cleaning. Patient compliance can also be monitored as the data are recorded.

A study carried out on children showed extraordinary possibilities of toothbrush application via smartphones. Forty-nine children received a manual toothbrush with an integrated sensor. Twenty-six children were randomly assigned to the test group and were given a smartphone app to help them visualize how they were cleaning and reward them according to the duration of tooth brushing. Plaque and gingival index were recorded at baseline, 6 and 12 weeks. At the recalls, the test group showed statistically significantly better oral health indices than the control group [18].

The duration of this pilot study (2 weeks) is in line with previous tooth brushing studies, which vary between 2 and 4 weeks [19,20,21]. The benefit of a shorter duration is that subject compliance during this time frame is more likely [20,21,22]. The sample size of 20 participants, although comparable to other studies, was rather small [20,21]. Nevertheless, a previous study with the same sample size and similar duration examined toothbrushes equipped with and without lithium batteries (active and inactive ionic toothbrushes), finding a statistical significance in the reduction of plaque scores and bleeding index in the active ionic toothbrush group [22]. However, the current study does present some limitations. There was no precise on-site control of how the app was applied and whether the participants adhered to the protocol, such as brushing their teeth twice a day. Gingival indices such as the gingival bleeding index or bleeding on probing were not recorded. A plaque index only gives insight to the brushing efficacy at the moment. A full follow-up study could benefit from having the control group use a smartphone timer when cleaning their teeth, as this would close the motivational gap of smartphone usage and help if the app’s localization feature or rather just the conscious measurement of time brings the most advantage to the user. Digitalization has become an intrinsic part of our lives and will continue to change current standard operating procedures in medicine and dentistry.

## 5. Conclusions

In summary, the pilot study showed that plaque index changes between the intervention group and the control group were not statistically different, however, the test group did enjoy a slight improvement in plaque removal over the control group. The user-friendliness of the app was determined to be moderate by the questionnaire, as only about half of the study participants were convinced of its effectiveness or would recommend it to others. The main reason for this was the inaccuracy of the toothbrush position detection and the time-consuming positioning of the tools for using the app. New technologies will further simplify some processes and their integration into working practice.

## Appendix A. The Questionnaire Translated from German into English Given to the Participants

Questionnaire
Please check the appropriate box or write your answer next to the Question.
1. (a) With which type of toothbrush do you brush your teeth at home?
manual toothbrush sonic toothbrush rotating toothbrush other
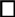
(b) Which model have you been using ?
2. Was the app easy to understand?
Yes No
3. If it was difficult to understand, in what way?

4. Did you have the impression that the app gave you a better cleaning result?
Yes No
5. Could you imagine using the app in the future?
Yes No
6. Why?
7. Would you recommend the app?
Yes No
8. Did use of the app bring about any changes in the way that you clean your teeth?
Yes No
9. What change(s) did you make?
10. Did you have any problems using the app?
Yes No
11. If so, what were these problems?
12. Do you have any other comments about the Oral-B app?
